# Untapped Endophytic Colonization and Plant Growth-Promoting Potential of the Genus *Novosphingobium* to Optimize Rice Cultivation

**DOI:** 10.1264/jsme2.ME16112

**Published:** 2017-02-21

**Authors:** Chakrapong Rangjaroen, Rungroch Sungthong, Benjavan Rerkasem, Neung Teaumroong, Rujirek Noisangiam, Saisamorn Lumyong

**Affiliations:** 1 Microbiology Division, Department of Biology, Faculty of Science, Chiang Mai UniversityChiang Mai 50200Thailand; 2 Department of Agricultural Management Technology, Faculty of Science and Technology, Phranakhon Rajabhat UniversityBangkok 10220Thailand; 3 Infrastructure and Environment Research Division, School of Engineering, University of GlasgowGlasgow G12 8LTUnited Kingdom; 4 Lanna Rice Research and Cultural Centre, Faculty of Agriculture, Chiang Mai UniversityChiang Mai 50200Thailand; 5 School of Biotechnology, Institute of Agricultural Technology, Suranaree University of TechnologyNakhonratchasima 30000Thailand; 6 National Bureau of Agricultural Commodity and Food Standards, Ministry of Agriculture and CooperativesBangkok 10900Thailand

**Keywords:** *Novosphingobium*, rice, diazotrophic bacteria, endophytic colonization, plant growth promoter

## Abstract

With the aim of searching for potent diazotrophic bacteria that are free of public health concerns and optimize rice cultivation, the endophytic colonization and plant growth-promoting activities of some endophytic diazotrophic bacteria isolated from rice were evaluated. Among these bacteria, the emerging diazotrophic strains of the genus *Novosphingobium* effectively associated with rice plant interiors and consequently promoted the growth of rice, even with the lack of a nitrogen source. These results suggest that diazotrophic *Novosphingobium* is an alternative microbial resource for further development as a safe biological enhancer in the optimization of organic rice cultivation.

Rice (*Oryza* spp.) is an important crop for human nourishment and is grown worldwide. The cultivation of commercial rice often requires nitrogen fertilizers in order to maintain and increase rice yields. However, traditional agricultural practices for the cultivation of local rice for household consumption (*e.g.*, without chemical input) also sustain reasonable rice yields each year (10, 13). The sustainability of rice yields involves various environmental factors, particularly soil-atmosphere nutrient cycles by beneficial microbes that occur in the rhizospheres of rice (6, 7, 13–15). The benefits of plant-microbe interactions are well-known factors that promote the growth of diverse rice cultivars to reach sustainable rice yields (6, 7).

Nitrogen-fixing microbes (so-called “diazotrophs”) offer significant benefits in the optimization of rice cultivation because they fix atmospheric nitrogen and supply usable forms of nitrogen to rice plants (14, 15). With the exception of obligate symbiotic diazotrophs (*e.g.*, *Rhizobium*), many other diazotrophs fix an unlimited amount of nitrogen in the free-living and endophytic phases (*e.g.*, *Klebsiella* and *Sphingomonas*) (14, 17). Attentive selection is essential for the further implementation of unlimited nitrogen-fixing microbes in rice cultivation because some of these microbes have been identified as health risks for farmers (*e.g.*, *Klebsiella pneumoniae*).

We recently revealed the nitrogen-fixing potential of endophytic diazotrophic bacteria isolated from the plant tissues of many rice landraces grown in northern Thailand (14). These endophytic diazotrophic bacteria belonged to diverse bacterial taxa, while some exhibited relatively high nitrogenase activities in the free-living and endophytic phases. In the present study, we aim to screen for potent diazotrophs among our previous list of endophytic diazotrophic bacteria, which are free of public health concerns and enhance rice growth with the lack of a nitrogen input. We selected some distinctive strains of endophytic diazotrophic bacteria from our previous study (14) listed in Table 1 for further evaluations of their taxonomy, endophytic colonization, and plant growth-promoting activities.

The classification of the selected strains was amended by a comparison of their 16S rRNA gene sequence data to those in the publicly available database in EzTaxon (www.ezbiocloud.net). All related sequences were selected, consequently aligned with MUSCLE, and used to construct the phylogenetic tree by MEGA7 (www.megasoftware.net). Among the selected strains, a representative from each taxonomic cluster was selected for labeling with red and green fluorescence tracks using a cloning technique. The pBZ1::dsRed2 (11) and pRK404 (9) plasmids were constructed and served as red and green fluorescence tracks, respectively. Each plasmid was introduced into *Escherichia coli* S17-1 (*pro recA* RP4-2(Tc^s^::Mu) (Km^s^::Tn*7*); Mob^+^) by an electroporation protocol described by Noisangium *et al.* (9). The plasmids were extracted from the obtained *E. coli* S17-1 transformant and purified with the QIAprep Spin Miniprep kit (QIAGEN, USA). The purified plasmids were introduced into the competent cells of our selected strains, using the same electroporation protocol described above (9). A modified method was performed in order to prepare competent cells from our selected strains (Supplemental Materials, Method S1). Fluorescence-labeled strains were used in further evaluations.

The commercial Thai Jasmine rice cultivar (*Oryza sativa* var. KDML 105) served as a mutual plant in the present study, and its rice seeds were surface sterilized and allowed to germinate on modified semisolid (MS) medium in a 50-mL test tube, following the protocol described elsewhere (3). Experiments were conducted either in the presence or absence of a water-soluble nitrogen input (NH_4_NO_3_). Therefore, MS medium supplemented with NH_4_NO_3_ at a final concentration of 0.2 mM served as the N-available treatment, and non-supplemented MS medium acted as the N-free treatment. After germinating for 3 d, the seedlings were inoculated with 1 mL of each fluorescence-labeled strain (at a cell density of ~10^7^ cells mL^−1^) and then allowed to grow at 27°C under the light-dark alternation (16 h in the light and 8 h in the dark). Colonized rice plant tissues were sampled and used for surface sterilization with a protocol described previously (14), and this was followed by cross-sectioning at a thickness of 100 μm using Microm HM 650V (Thermo Scientific, USA).

The endophytic colonization of fluorescence-labeled strains was tracked and observed with an inverted fluorescence microscope (Olympus IX51, USA). Viable counts of bacterial cells colonized at different rice plant interiors were also confirmed (Method S2). The heights of rice shoots represented a growth indicator of rice in this study; they were measured and calculated in percentages of increased heights over those measured in the absence of the bacterial inoculum (control). Any means±SDs or SEs derived from at least triplicate experiments were statistically compared using an analysis of variance (ANOVA) in SPSS 22.0 software (SPSS, Chicago IL, USA). Statistical results are indicated with *F*-distribution values, degrees of freedom, and significance (*P*) levels.

The phylogenetic reclassification with the Maximum Likelihood method revealed that potent endophytic diazotrophic bacteria belonged to the families *Burkholderiaceae*, *Enterobacteriaceae*, and *Sphingomonadaceae* (Fig. S1). The closely related phylogenetic species of these bacteria listed in Table 1 were different to our previous classification (14). Among all isolates, three were members of the genus *Novosphingobium* (formerly classified in the genus *Sphingomonas* [14]) and closely related to *N. sediminicola* HU1-AH51^T^. However, the % identities of these *Novosphingobium* isolates to their closest species were relatively small (96.98–97.10%), suggesting the high possibility of their species novelty. This range of % identities was similar to a recently released species, *N. oryzae* that was proposed as a novel species with ≤97.2% identities to other species in the same genus (18). Since the establishment of the genus *Novosphingobium* in 2001 (16), a few of its members have been identified as endophytes of rice (*e.g.*, *N. fluoreni* from rice seeds [4] and *N. oryzae* from rice roots [18]). Previous studies revealed the plant growth-promoting potential of the genus *Novosphingobium* (1, 14, 18), while *N. nitrogenifigens* is so far the first and only diazotrophic strain of this genus (1).

We selected *Novosphingobium* sp. PS5, *Klebsiella* sp. SS2, and *Burkholderia* sp. SS5 as a representative of each genus for further comparative studies of their endophytic colonization and plant growth-promoting potentials for optimizing rice cultivation. The endophytic colonization of rice interiors by *Novosphingobium* sp. PS5 is shown in Fig. 1. At 1 d after inoculation (DAI), strain PS5 colonized root hairs by forming microcolonies on the surfaces of epidermal cells, particularly around the emerging sites of lateral roots. Consequently, strain PS5 invaded the plant interiors at the parenchymatous root cortex, from which it migrated through the intercellular and intracellular spaces of the rice shoots, vascular bundles of leaves, and lateral roots (at 7 DAI). Similar colonizing patterns were observed with *Klebsiella* sp. SS2 (Fig. S2) and *Burkholderia* sp. SS5 (Fig. S3). We confirmed the abundant occupancies of the test bacteria colonized within the root and shoot interiors of rice with their viable counts (Table S1). The results obtained suggested that rice roots were an optimal habitat, housing large numbers of every test bacteria, while *Novosphingobium* sp. PS5 exhibited the highest abundance in the colonization of root and shoot interiors. The greater % of the increased height of rice shoots also corresponded to the abundant occupancy within the rice plant interiors of strain PS5 (Fig. 2).

The mechanisms by which diazotrophic *Novosphingobium* invaded into and migrated within the rice interiors have yet to be elucidated. The plant rhizosphere and root exudates are considerable boundaries that offer an optimal ecological niche for synergistic interactions between plants and soil-dwelling microbes. Wound sites and openings that occur naturally by elongation and differentiation during rice growth and development are recognized as potential routes for plant invasion by endophytic diazotrophic bacteria (5, 7). This may support the abundance of bacterial cells that initially colonized these wound sites and openings in rice roots. The possession of cellulolytic enzymes has been proposed as a biochemical tool that endophytic diazotrophic bacteria use to invade the plant interior (12). The bacterial strains tested in the present study also exhibited the ability to form at least two cell wall lytic enzymes, *i.e.*, cellulase, pectinase, and chitinase (14). Further studies on how these enzymes are expressed and function during the invasion and colonization of plant interiors will contribute to a better understanding of the biological processes involved in plant-microbe interactions. Based on our results, rice seedlings were healthy and there were no apparent disease symptoms detected after endophytic colonization by any bacteria tested, which supports mutual interactions between them and rice plants.

It is conceivable that nitrogen input in the cultivation of rice may either interfere with the community of diazotrophic bacteria or suppress their nitrogen-fixing activities (8, 12). Furthermore, endophytic colonization is a mechanism by which diazotrophic bacteria protect themselves and maintain their nitrogen-fixing activities from surrounding stresses (*e.g.*, oxygen-induced damage of nitrogenase, competition with other rhizobacteria) (2). Our previous findings showed that all bacteria tested were potent diazotrophs with the ability to fix atmospheric nitrogen either in the absence or presence of a water-soluble nitrogen input, and either in their free-living phase or in association with their mutual rice plant (14). Diazotrophic *Novosphingobium* exhibited the most distinctive endophytic colonization and plant growth-promoting potentials, as well as the free of public health concern, when compared to the other bacteria tested. We conclude that the diazotrophic *Novosphingobium* would be a promising microbial resource for further development as an efficient and safe biofertilizer for optimizing the organic rice cultivation.

## Supplemental material



## Figures and Tables

**Fig. 1:**
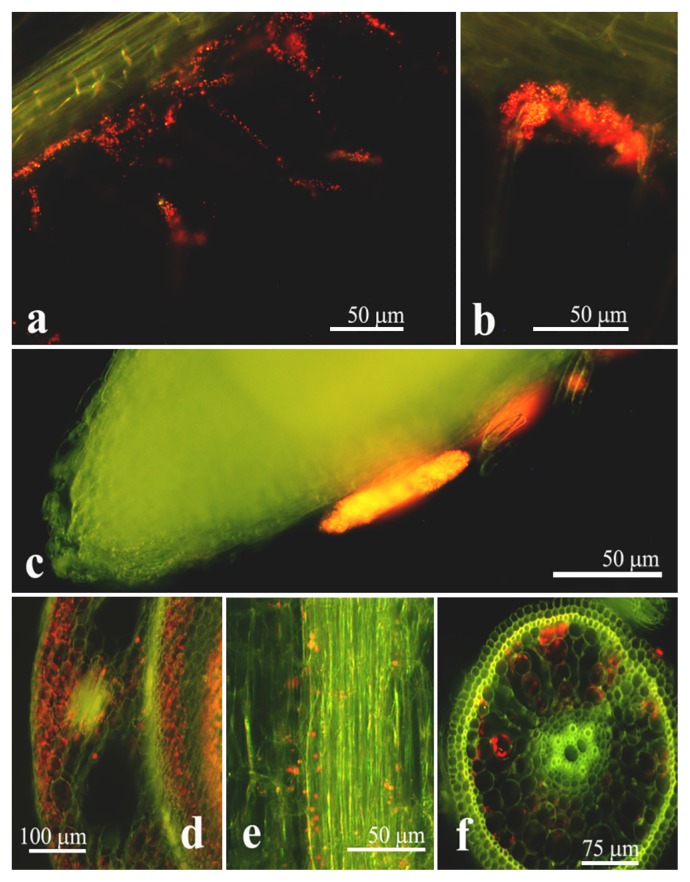
Rice plant-colonizing pattern of *Novosphingobium* sp. PS5. Red particles in micrographs represent the red fluorescent cells of strain PS5. The surface colonization of root hairs (a, b) and formation of microcolonies on epidermal cells of the root (c) by strain PS5 were observed 1 d after inoculation (DAI). Interior colonization on the stem (d), vascular bundles in leaves (e), and a new root (f) was observed at 7 DAI.

**Fig. 2:**
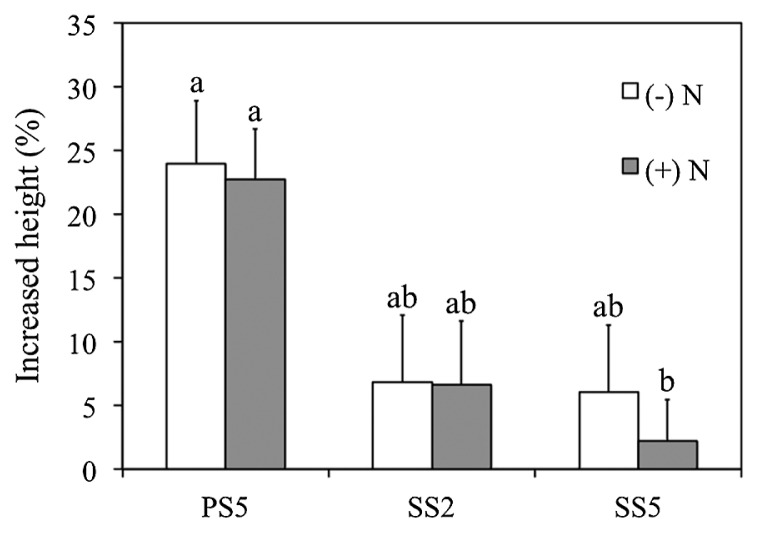
Impacts of different bacterial strains on the growth of rice plants. Three different bacterial strains: *Novosphingobium* sp. PS5, *Klebsiella* sp. SS2, and *Burkholderia* sp. SS5 were compared, and *Oryza sativa* var. KDML 105 was used as the mutual host plant. The graph is plotted with means and error bars of SE derived from 15 rice seedlings grown in the absence (−) and presence (+) of a nitrogen input (N). In the presence of N, 0.2 mM NH_4_NO_3_ was applied (see the text for details). The heights of rice shoots were measured 14 d after the inoculation with bacteria, and increased heights were calculated in percentages based on comparisons with the average height of rice shoots derived from the control without bacteria. Different lower case letters refer to the significance of differences in means by a one-way ANOVA with Tukey’s *post hoc* test (*F*_(5, 84)_=4.057, *P*=0.05).

**Table 1:** Distinctive strains of endophytic diazotrophic bacteria used in this study.

Bacterial strain^a^	GenBank accession no.	Closest species	Identity (%)^b^	Different nt/Total nt^b^
PR1	JX083379	*Burkholderia kururiensis* JCM 10599^T^	100.00	0/1489
PS5	JX083381	*Novosphingobium sediminicola* HU1-AH51^T^ 9	7.03	40/1346
SR6	JX083384	*Novosphingobium sediminicola* HU1-AH51^T^	97.10	35/1207
SS2	JX083385	*Klebsiella pneumoniae* subsp. *pneumoniae* DSM 30104^T^	99.56	6/1351
SS5	JX083386	*Burkholderia kururiensis* JCM 10599^T^	100.00	0/1325
SS6	JX083387	*Novosphingobium sediminicola* HU1-AH51^T^	96.98	41/1356

a The origins of these bacterial strains are shown in (13).

b Results are taken from the alignment search tool of EzTaxon (www.ezbiocloud.net, search on 26 May 2016).
