# Supplementary material for: Untapped Endophytic Colonization and Plant Growth-Promoting Potential of the Genus Novosphingobium to Optimize Rice Cultivation
Source: Microbes Environ. 2017 Feb 21;32(1):84–7. doi: 10.1264/jsme2.ME16112 (PMC5371080; doi:10.1264/jsme2.ME16112)

Supplemental material

Untapped endophytic colonization and plant growth-promoting potential of the genus *Novosphingobium* for optimizing rice cultivation

Chakrapong Rangjaroen[1,2], Rungroch Sungthong[3], Benjavan Rerkasem[4], Neung Teaumroong[5], Rujirek Noisangiam[5], Saisamorn Lumyong[6]*

[1]Microbiology Division, Department of Biology, Faculty of Science, Chiang Mai University, Chiang Mai 50200, Thailand

[2]Department of Agricultural Management Technology, Faculty of Science and Technology, Phranakhon Rajabhat University, Bangkok 10220, Thailand

[3]Infrastructure and Environment Research Division, School of Engineering, University of Glasgow, Glasgow G12 8LT, United Kingdom

[4]Lanna Rice Research and Cultural Centre, Faculty of Agriculture, Chiang Mai University, Chiang Mai 50200, Thailand

[5]School of Biotechnology, Institute of Agricultural Technology, Suranaree University of Technology, Nakhonratchasima 30000, Thailand

[6]National Bureau of Agricultural Commodity and Food Standards, Ministry of Agriculture and Cooperatives, Bangkok 10900, Thailand

***Corresponding author:** Saisamorn Lumyong; E-mail: scboi009@gmail.com; Tel: +66 (0) 53 941946; Fax: +66 (0) 53 941949

Running title: Rice growth-promoting *Novosphingobium*

Method S1. A modified method for preparation of competent cells.

A bacterial colony was transferred into 25 mL of N-free broth (3) and incubated overnight at 28°C with shaking at 120 rpm. One mL of the culture broth with an optical density ($OD_{600\ nm}$) of 0.6 was inoculated into 150 mL Luria-Bertani (LB) broth (compositions per L: 10 g tryptone, 5 g yeast extract and 10 g NaCl) and incubated at the same condition mentioned above, till the OD_{600} reached to 0.3. The culture broth was then incubated on ice for 20 min, and the bacterial cells were collected by centrifugation at 2630 ×*g*, 4°C for 20 min. The collected cells were washed four times with cold 10% (v/v) glycerol and centrifugation at the same condition as described above. The washed cells were resuspended with 1 mL 25% (v/v) glycerol and preserved at -80°C till use.

Method S2. Quantification of bacterial viable counts after colonizing rice plant interiors.

The viable counts of the selected diazotrophic bacteria, strains PS5, SS2 and SS5 after colonizing the root and shoot interiors of rice seedlings for 3 and 14 days were quantified using triplicate rice seedlings. The rice seedlings were collected and divided into root and shoot parts, which were then washed with distilled water to remove attached bacteria on the rice surfaces. The root and shoot parts were surface sterilized following the protocol described elsewhere (13) and cut into small pieces. The cut plant tissues were ground using a sterile mortar, serially diluted, and spread on LB agar plates supplemented with tetracycline and gentamicin at the final concentration of 50 μg mL^{-1}. The seeded agar plates were incubated at 28ºC for 48 h. The appeared bacterial colonies were counted and reported as log CFU g^{-1} of plant tissue. Axenic rice seedlings were served as controls.

Table S1 Viable count of diazotrophic bacteria in different plant interiors of rice seedlings.

Bacterial strain	Viable count (log CFU g^{-1} plant tissue) of bacteria[a]			
	3 DAI		14 DAI	
	Root[b]	Shoot[c]	Root[d]	Shoot[e]
PS5	4.18 ± 0.83a	3.58 ± 0.46a	4.39 ± 0.81a	2.64 ± 0.55a
SS2	3.30 ± 1.04a	2.96 ± 0.18a	1.42 ± 0.67b	1.32 ± 0.30a
SS5	3.97 ± 0.23a	3.17 ± 0.54a	3.62 ± 0.46a	1.53 ± 0.90a

[a]The bacterial viable counts that colonized different plant interiors (roots and shoots) of rice seedlings were quantified at 3 and 14 days after inoculation (DAI) of bacterial culture. Different lower case letters refer to the statistical difference of means compared by one-way ANOVA with Tukey's *post hoc* test between different bacterial strains tested, [b]$F_{(2, 6)} = 1.049$, $P = 0.407$, [c]$F_{(2, 6)} = 1.671$, $P = 0.265$, [d]$F_{(2, 6)} = 16.272$, $P = 0.004$, and [e]$F_{(2, 6)} = 3.744$, $P = 0.088$.

Fig. S1. Unrooted maximum likelihood phylogenetic tree of nearly full-length 16S rRNA gene sequences showing the relationship among the nitrogen-fixing bacteria. Bootstrap analysis was based on 1000 replicates, and scale bar represents 0.02 nucleotides substitution. The accession numbers of the sequences are indicated in the parentheses.

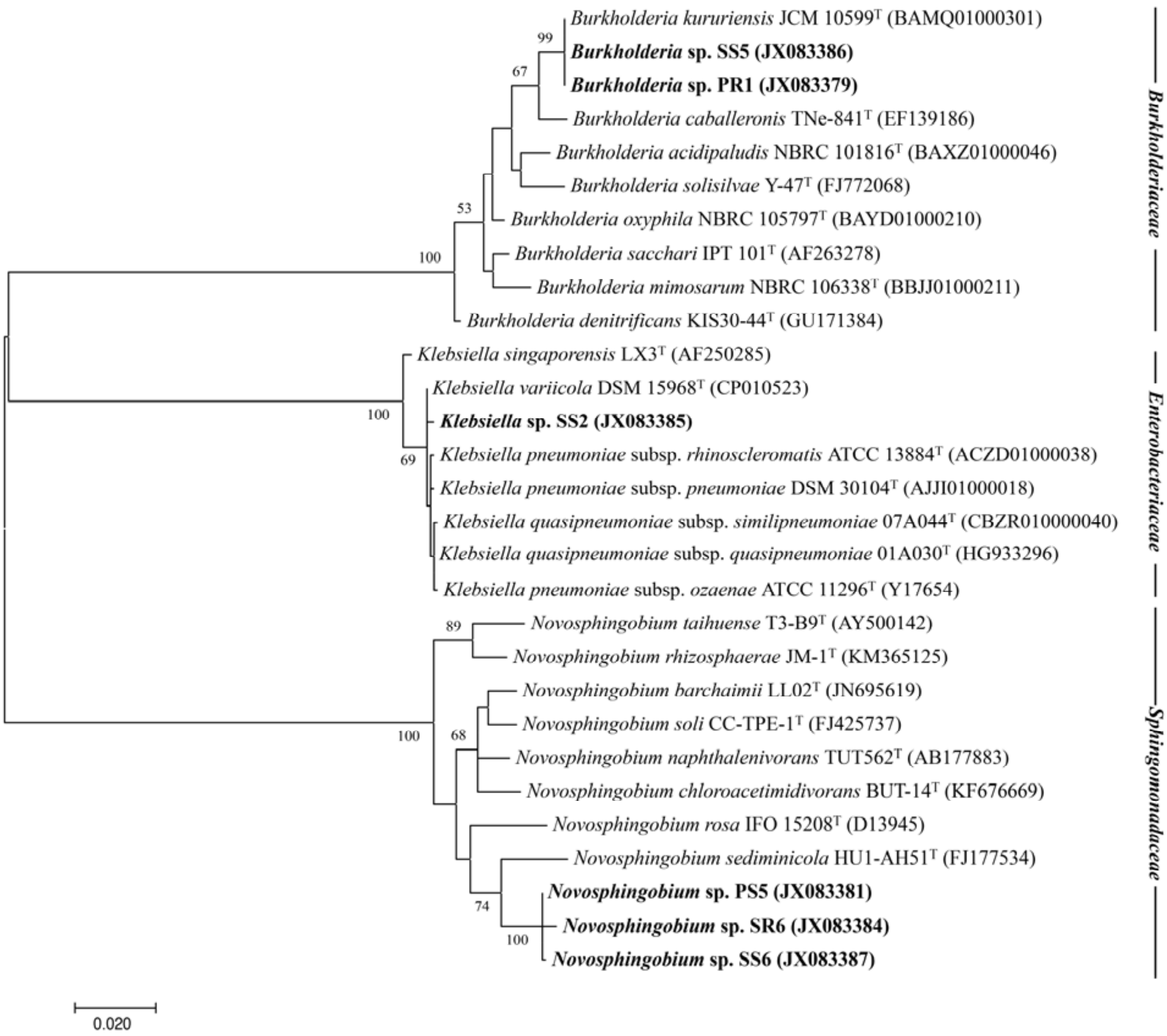

Fig. S2. Rice plant surfaces and interiors colonized by *Klebsiella* sp. SS2. Red particles in micrographs represent the red fluorescent cells of strain SS2. Surface colonization of root hair (a, b) and formation of microcolony on epidermal cells of root (c) by strain SS2 were observed at 1 day after inoculation (DAI). Interior colonization on stem (d), vascular bundle in leaf (e), and new root (f) was observed at 7 DAI.

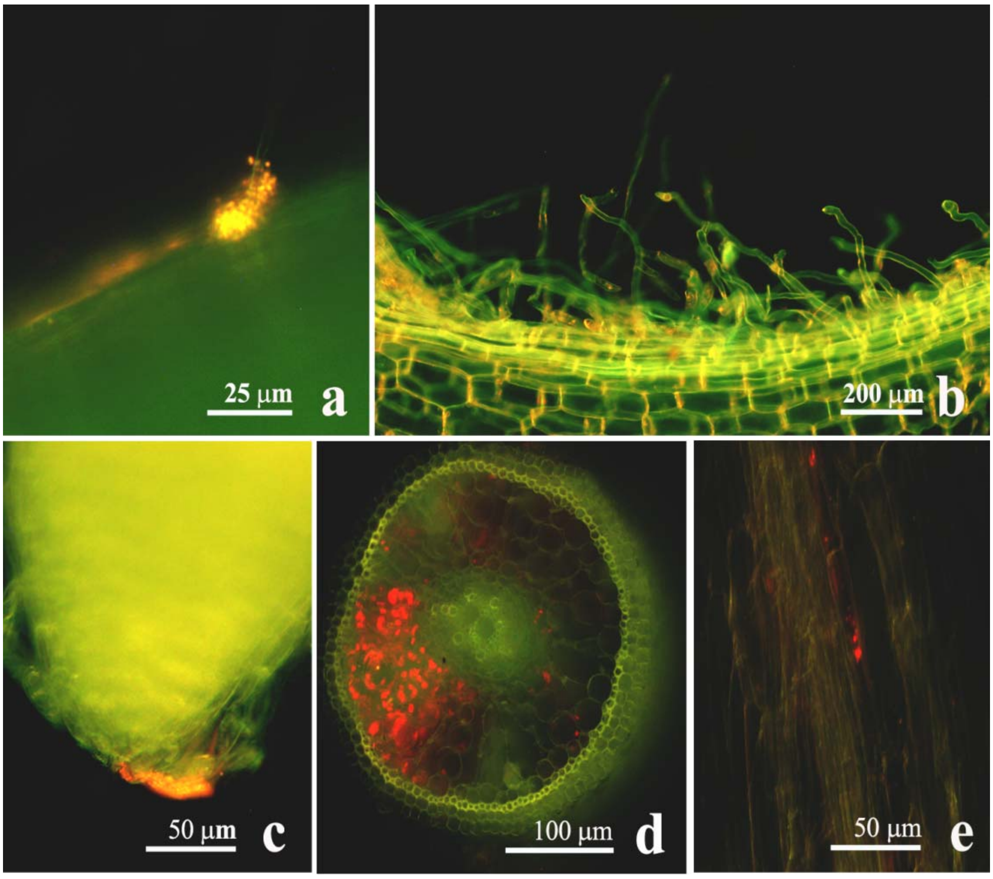

Fig. S3. Rice plant surfaces and interiors colonized by *Burkholderia* sp. SS5. Green particles in micrographs represent the green fluorescent cells of strain SS5. Surface colonization of root hair (a, b) and formation of microcolony on epidermal cells of root (c) by strain SS5 were observed at 1 day after inoculation (DAI). Interior colonization on stem (d), vascular bundle in leaf (e), and new root (f) was observed at 7 DAI.

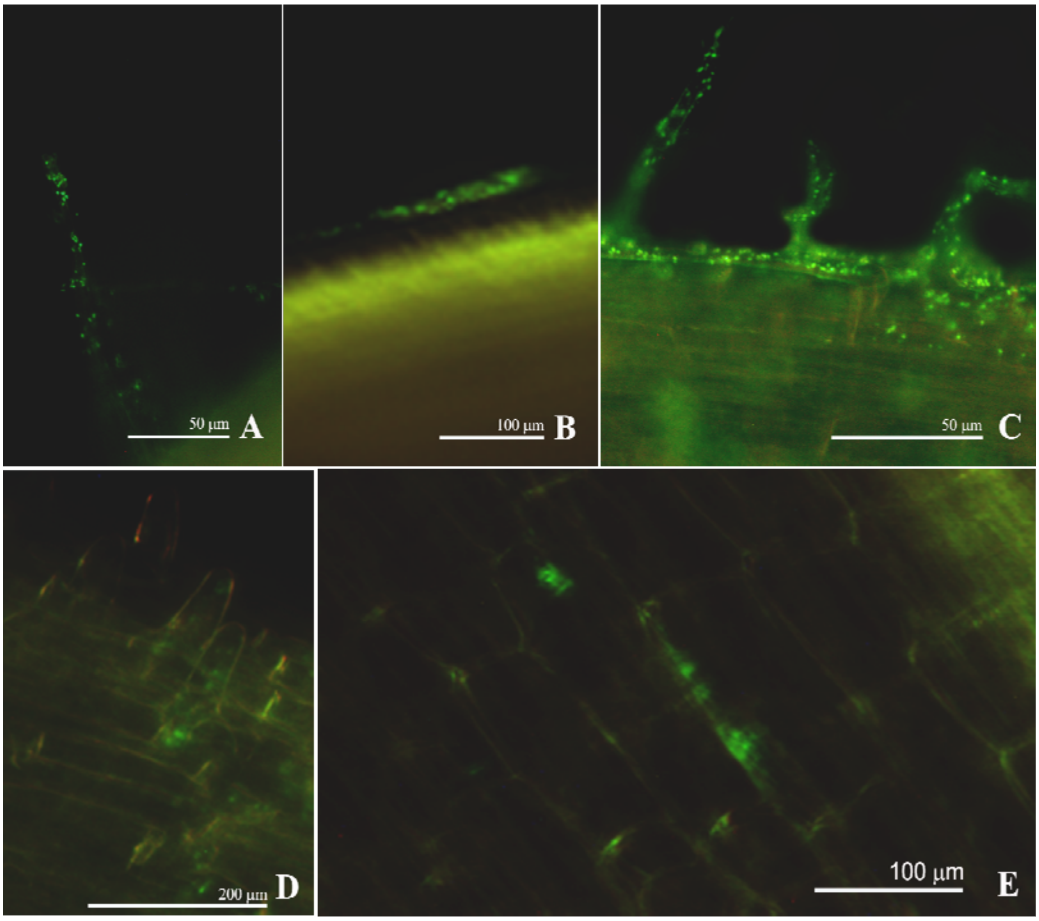

Supplement: Supplementary file 1 [file 32_84_s1.pdf]
